# DNA-based quantification and counting of transmission stages provides different but complementary parasite load estimates: an example from rodent coccidia (*Eimeria*)

**DOI:** 10.1186/s13071-021-05119-0

**Published:** 2022-02-04

**Authors:** Víctor Hugo Jarquín-Díaz, Alice Balard, Susana Carolina Martins Ferreira, Vivian Mittné, Julia Mari Murata, Emanuel Heitlinger

**Affiliations:** 1 Institute for Biology, Department of Molecular Parasitology, Humboldt University Berlin (HU), Philippstr. 13, Haus 14, 10115 Berlin, Germany; 2 Leibniz-Institut Für Zoo- Und Wildtierforschung (IZW), im Forschungsverbund Berlin e.V., Alfred-Kowalke-Straße 17, 10315 Berlin, Germany; 3 Division of Computational Systems Biology, University of Vienna, Althanstr. 14, 1090 Wien, Austria; 4 Present Address: Experimental and Clinical Research Center, jointly operated by Charité–Universitätsmedizin Berlin and the Max Delbrück Center for Molecular Medicine, Charité Campus Berlin Buch, Lindenberger Weg 80, 13125 Berlin, Germany

**Keywords:** *Eimeria*, Oocysts, qPCR, Parasite load, Resistance

## Abstract

**Background:**

Counting parasite transmission stages in faeces is the classical measurement to quantify “parasite load”. DNA-based quantifications of parasite intensities from faecal samples are relatively novel and often validated against such counts. When microscopic and molecular quantifications do not correlate, it is unclear whether oocyst counts or DNA-based intensity better reflects biologically meaningful concepts. Here, we investigate this issue using the example of *Eimeria ferrisi* (Coccidia), an intracellular parasite of house mice (*Mus musculus*).

**Methods:**

We performed an infection experiment of house mice with *E. ferrisi*, in which the intensity of infection correlates with increased health impact on the host, measured as temporary weight loss during infection. We recorded the number of parasite transmissive stages (oocysts) per gram of faeces (OPG) and, as a DNA-based measurement, the number of *Eimeria* genome copies per gram of faeces for 10 days post-infection (dpi). We assessed weight loss relative to the day of experimental infection as a proxy of host health and evaluated whether DNA or oocyst counts are better predictors of host health.

**Results:**

Absolute quantification of *Eimeria* DNA and oocyst counts showed similar but slightly diverging temporal patterns during 10 dpi. We detected *Eimeria* DNA earlier than the first appearance of oocysts in faeces. Additionally, *Eimeria* OPGs within each dpi did not explain parasite DNA intensity. Early dpi were characterized by high DNA intensity with low oocyst counts, while late infections showed the opposite pattern. The intensity of *Eimeria* DNA was consistently a stronger predictor of either maximal weight loss (1 value per animal during the infection course) or weight loss on each day during the experiment when controlling for between-dpi and between-individual variance.

**Conclusions:**

*Eimeria ferrisi* oocyst counts correlate weakly with parasite intensity assessed through DNA quantification. DNA is likely partially derived from life-cycle stages other than transmissive oocysts. DNA-based intensities predict health outcomes of infection for the host more robustly than counts of transmissive stages. We conclude that DNA-based quantifications should not necessarily require validation against counts of transmissive stages. Instead, DNA-based load estimates should be evaluated as complementary sources of information with potential specific biological relevance for each host-parasite system.

**Graphical Abstract:**

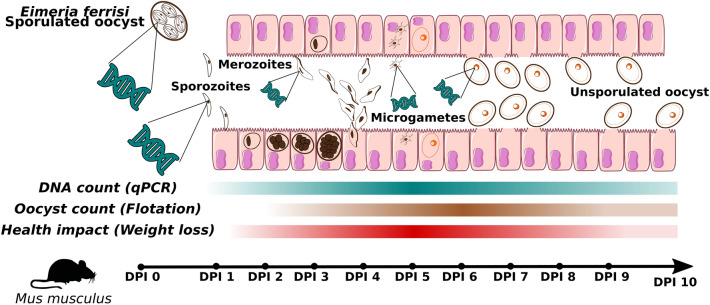

**Supplementary Information:**

The online version contains supplementary material available at 10.1186/s13071-021-05119-0.

## Background

In the last decade, ‘molecular techniques’ based on the quantification of nucleic acids (DNA for eukaryotic parasites) are increasingly complementing or even replacing classical coprological estimates of parasite load. However, such DNA-based techniques are often thought to still require validation against a classical standard procedure, such as the counting of parasite reproductive and transmissive stages [1–3].

Asexual stages of many protozoans replicate within the tissues of their host before commitment to sexual stage differentiation and eventual fertilization in the same or different hosts. For monoxenous intracellular parasites like Coccidia, the count of their reproductive stages (oocysts) has been used to measure parasite load, as in the context of estimating host resistance [4–7]. The estimation of tissue load is complex for protozoa, especially with classical histological techniques. Thus, oocysts floated from faeces are the firmly established standard for quantification of Coccidia [8, 9].

Qualitative and quantitative techniques for nucleic acid detection (PCR and quantitative PCR [qPCR]) have been used on tissue or faeces of economically relevant host species (e.g. poultry) and animal models (e.g. rodents) [10–16]. It is, however, still unclear how oocyst counts and DNA-based quantification of Coccidia correlate along the course of infection and whether both can be interpreted similarly or differently in terms of their biological meaning for host and parasite.

*Eimeria ferrisi* is a species of Coccidia that specifically parasitizes the house mouse, *Mus musculus* [17]. The sporozoites of *E. ferrisi* infect the surface of villar epithelial cells in the caecum [18, 19]. The asexual expansion of the parasite comprises three cycles of merogony before commitment to sexual replication, which results in the formation of gamonts. The first generation of schizonts of *E. ferrisi* can be observed in tissue at 24 h post-infection). The second generation of merozoites is distinguishable in tissue at 36 h PI and the third at 72 h PI [18, 20]. Electron microscopy observations indicate that at 3 days post-infection (dpi) individual parasitized cells have already released merozoites onto neighbouring cells [21]. After the resulting prepatent period of 4 days, sexual gametocytes can be observed from 4 to 6 dpi, during a patent period of 3–4 days. A peak of oocysts shedding is reached at 6 dpi [18, 22]. This life-cycle is shorter compared to that of other *Eimeria* house mice (e.g. *E. vermiformis* or *E. falciformis*) [19].

*Eimeria ferrisi* infections cause diarrhoea and weight loss through tissue damage during the asexual and sexual reproduction of the parasite. Tissue damage is considered to be most severe during the patent period [21]. *Eimeria ferrisi* has been reported to be the most prevalent *Eimeria* species in natural populations of *M. musculus* [16]. Disease tolerance does not impact (i.e. reverse, as in *E. falciformis* infections) the quantitative effect of *E. ferrisi* on the health of its host, meaning that the amount of oocysts detected during an infection correlates positively with the severity of the impact on host health during the patent period [7].

The aim of the present investigation was to understand how classical coprological and DNA-based methods for parasite quantifications correlate in Coccidia. To this end, we established a real-time qPCR method for absolute quantification of *E. ferrisi* DNA in host faeces. Our results show that the amount of faecal DNA correlates only weakly with oocyst counts. Finally, we report that the intensity of DNA in faeces is a better predictor for parasite impact on host health.

## Methods

### Animal husbandry

Twenty-two house mice (*M. musculus*) of the “wild-derived” inbred strains SCHUNT, STRA, BUSNA and PWD as well as F1 inter-strain crosses [7, 23–25] were obtained from the Institute of Vertebrate Biology of the Czech Academy of Science in Studenec (licence: 61974/2017-MZE-17214). The age of the mice at infection ranged from 9 to 19.43 weeks and weight at start of experiments ranged from 15.16 to 28.95 g (Additional file 1: Table S1). All mice were acclimatized to the animal experiment facilities of the Humboldt University for at least 1 week before infection. Mice were housed in individual cages and provided with food and water ad libitum during the experiment.

### Parasites and inoculum preparation

*Eimeria ferrisi* Brandenburg64 (hereafter E64) was isolated from the faeces of a wild *M. musculus domesticus* mouse captured in Brandenburg, Germany in 2016 and identified by microscopical description and molecular amplification of the 18S ribosomal RNA and cytochrome* c* oxidase (COI) markers [16]. E64 oocysts were produced by continuous passage in NMRI mice and sporulated as described previously [7]. Oocysts of the strain *E. falciformis* Bayer-Haberkorn [26, 27] were used for the generation of DNA standards.

### Experimental design

Mice were orally infected with 150 sporulated oocysts in 100 µl of phosphate-buffered saline (PBS 1×, pH  7.4) and monitored for 11 days. Weight was recorded daily, and a weight loss of 18% was defined as the humane endpoint at which animals had to be sacrificed (experiment license: 0431/17). At the end of the experiment, mice were euthanized by cervical dislocation. An average of 0.12 g of faeces (3–4 faecal pellets) from individual mice were collected daily, weighed, flash frozen in liquid nitrogen and later stored at − 80 °C for DNA extraction at a later time. The remaining faeces in the cage were collected on the day of infection (0 dpi) and daily from 3 to 10 dpi, weighed and maintained in potassium dichromate 2.5% (w/v) (K_2_Cr_2_O_7_) (Carl-Roth GmbH + Co. KG, Karlsruhe, Germany) at room temperature until further flotation.

### Flotation and quantification of oocysts

To eliminate the potassium dichromate, faecal material was washed three times with tap water, following which a saturated salt solution (specific gravity: 1.18–1.20) was added to the faecal samples and the samples vigorously mixed on a vortex to homogenize the material. The faecal suspension was left to stand for 10 min and then centrifuged (3234 *g*, 21 °C for 10 min) for oocyst flotation. The upper layer of the suspension was collected and washed with distilled water (1800 *g*, 21 °C for 10 min), and the resulting pellet was resuspended in 1 ml of 2.5% potassium dichromate. Flotation products were inspected microscopically for the presence of oocysts using a Leica® DM750 M light microscope with a 10×–40× objective (Leica Microsystems GmbH, Wetzlar, Germany). Floated oocysts were counted using a Neubauer chamber, and the results were expressed as oocysts per gram (OPG) of faeces.

### Mock sample preparation

Standard quantities of oocysts ranging from 10^0^ to 10^6^ were spiked into 1.2 g faeces from uninfected mice (*n* = 8). The oocysts were then homogenized with the faecal material prior to DNA extraction.

### DNA extraction

Genomic DNA (gDNA) from faeces collected in the infection experiment (*n* = 242), mock samples (*n* = 8) or flotation products (*n* = 19) was extracted using a NucleoSpin®Soil kit (Macherey-Nagel GmbH & Co. KG, Düren, Germany) following the manufacturer’s protocol with the following modifications: mechanical lysis of the sample was performed in the Precellys®24 high-speed benchtop homogenizer (Bertin Technologies, Aix-en-Provence, France) with the following lysis programme: 2 cycles of disruption at 6000 rpm for 30 s, with 15-s delay between cycles. For each sample, extraction was repeated once to maximize the DNA yield, and nucleic acids were eluted with 40 µL of TE buffer. The quality and integrity of the DNA were assessed using a full-spectrum spectrophotometer (NanoDrop 2000c; Thermo Fisher Scientific, Waltham, MA USA). Concentrations of double-stranded DNA were quantified using a Qubit® Fluorometer and the dsDNA BR (broad-range) Assay Kit (Thermo Fisher Scientific). DNA extracts were adjusted to a final concentration of 50 ng/µl with nuclease-free water (Carl-Roth GmbH + Co. KG) and stored at − 80 °C until further processing.

### Preparation of standardized quantities of oocysts

*Eimeria falciformis* and *E. ferrisi* DNA standards were generated from aliquots of 2 × 10^6^ and 2.1 × 10^6^ sporulated oocysts, respectively, counted as described above. Tenfold successive dilutions from the extracted gDNA were generated in nuclease-free water to represent a range from 10^0^ to 10^6^ oocysts. qPCR readings were produced by real-time qPCR assays (see section Real-time qPCR) of the serial dilutions. These assays were performed in three independent runs in triplicate for each diluted DNA.

For our model, the use of a mitochondrial marker has the advantage of increasing detection sensitivity due to multiple copies (approx. 180 per nuclear genome equivalent for *E. falciformis*; [27]). We expected eight *Eimeria* haploid nuclear genomes per oocyst and assumed that a fully sporulated oocyst would similarly provide an eightfold higher number of mitochondrial DNA copies than other haploid life-cycle stages. The final standard curve was generated by estimating a linear model predicting the log10-transformed genome copies by the cycle threshold (Ct) value using DNA extracted from fully sporulated oocysts. A single linear model accounting for the variation of the different qPCR platforms employed during the experiment was used in all further analyses to predict genome equivalents from the Ct values.

### Real-time qPCR

*Eimeria* DNA was quantified by qPCR amplification of a 140-bp fragment of the mitochondrially encoded COI gene (*COI*) using *Eimeria*-specific primers Eim_COI_qX_F 5ʹ-TGTCTATTCACTTGGGCTATTGT-3ʹ and Eim_COI_qX_R 5ʹ-GGA TCACCGTTAAATGAGGCA-3ʹ [16]. Each reaction contained 1× iTaq™ Universal SYBR® Green Supermix (Bio-Rad Laboratories, Hercules, CA USA), 400 nM forward and reverse primers and 50 ng template gDNA in a total reaction volume of 20 µl. Reactions were performed either in the ABI 7300 Real-Time PCR System (Applied Biosystems, Thermo Fisher Scientific, Foster City, CA, USA), the MasterCycler® RealPlex2 machine (Eppendorf, Hamburg, Germany) or the CFX96™ Touch System (Bio-Rad Laboratories) for DNAs from mock samples, floated oocysts and faecal DNA derived from the infection experiment. For all PCR systems, cycling conditions were: an initial denaturation at 95 °C for 2 min; 40 cycles of denaturation at 95 °C/15 s, annealing at 55 °C/15 s and extension at 68 °C/20 s, with data collection at the end of each cycle. Melting curve analysis was included to discard primer dimer formation and non-specific amplification: after the last amplification cycle, the temperature was increased from 65 °C to 95 °C with 0.5 °C increments and 3 s per step. Amplifications were performed in triplicate, and each run included a non-template control (NTC). Melting curves were analysed blindly (for dpi) for the presence of distinct “*Eimeria* products” and PCR artefacts. Samples with all three replicates showing the melting temperature (Tm) in the range 74.1 °C  ± 1.78 °C (observed on positive controls; Additional file 2: Fig. S1) were labelled as “qPCR positive”, samples with only one or two of the triplicates showing peaks were designated as negative samples. For qPCR-negative samples, genome copies per gram of faeces were set to 0.

### Data analysis and statistics

Genome copies were predicted from the linear model fitted for the respective standard curve. The predictions present an estimate of the *Eimeria* genome copies based on the Ct and taking into consideration the qPCR platform employed. Differences between models with or without cycler as a predictor were assessed using likelihood ratio tests (LRT; R function “lrtest” from package “lmtest” [28]). The results from floated oocyst samples and faecal spiked samples were expressed as genome copies for direct comparison with the standard curve results. For further analysis using faecal DNA derived from the infection experiment, the results were reported as genome copies per gram of faeces to compare with OPG counts. The arithmetic mean and standard deviation (SD) for each sample were calculated based on the prediction for triplicates.

A two-sided paired Wilcoxon signed-rank test was performed to evaluate whether there was a significant increase in the number of *Eimeria* genome copies per gram of faeces, in OPG counts and in the percentage of weight loss at each time point during the infection compared to dpi 0. Adjusted *P*-values were reported.

We predicted *Eimeria* genome copies per gram of faeces by OPG in a linear model. We compared a model including different intercepts and slopes for each dpi (an interaction effect of dpi with OPG) to the model without dpi.

To test whether OPG and *Eimeria* genome copies per gram of faeces and interaction between the two can be used to explain weight loss, we used two statistical approaches. With the first approach, we tested whether the maximum quantity of *Eimeria* DNA per gram of faeces predicts maximum weight loss better than the maximum number of OPG. We fitted a linear model predicting maximum relative weight loss as a response of maximum OPG shed and maximum DNA per gram of faeces, including an interaction effect between the two measures. With the second approach, we used a linear model after transformation in which the within-individual mean and within-dpi mean were subtracted from all observations (time and subject demeaned). This approach allows elimination of within-subject effects (changes associated with each individual that are time-invariant) and within-time effects (changes over time that affect all observations in the same way; time-variant variability) [29–31].

We used diagnostic plots of residuals to assess the fit of linear models. To test the significance of the marginal contribution of each parameter to the full model, each parameter was removed from the full model, and the difference between the full and reduced model was assessed using likelihood ratio tests (LRT; R function “lrtest” from package “lmtest” [28]). The global goodness of fitness was assessed by LRT to compare the full models with intercept-only models. To assess the relative contribution of each predictor to the variance explained by a linear model, we used the R package “relaimpo” version 2.2–3 [32] which is based on the LMG statistic [33].

Analyses were performed using R v.4.0.3 [34]. Figures were generated using the R package ggplot2 [35].

All the code and raw data required for the analysis is publicly available at https://github.com/derele/Eimeria_Quant.

## Results

### Absolute quantification of* Eimeria* DNA is robust for different DNA extractions on multiple amplification platforms

**Fig. 1:**
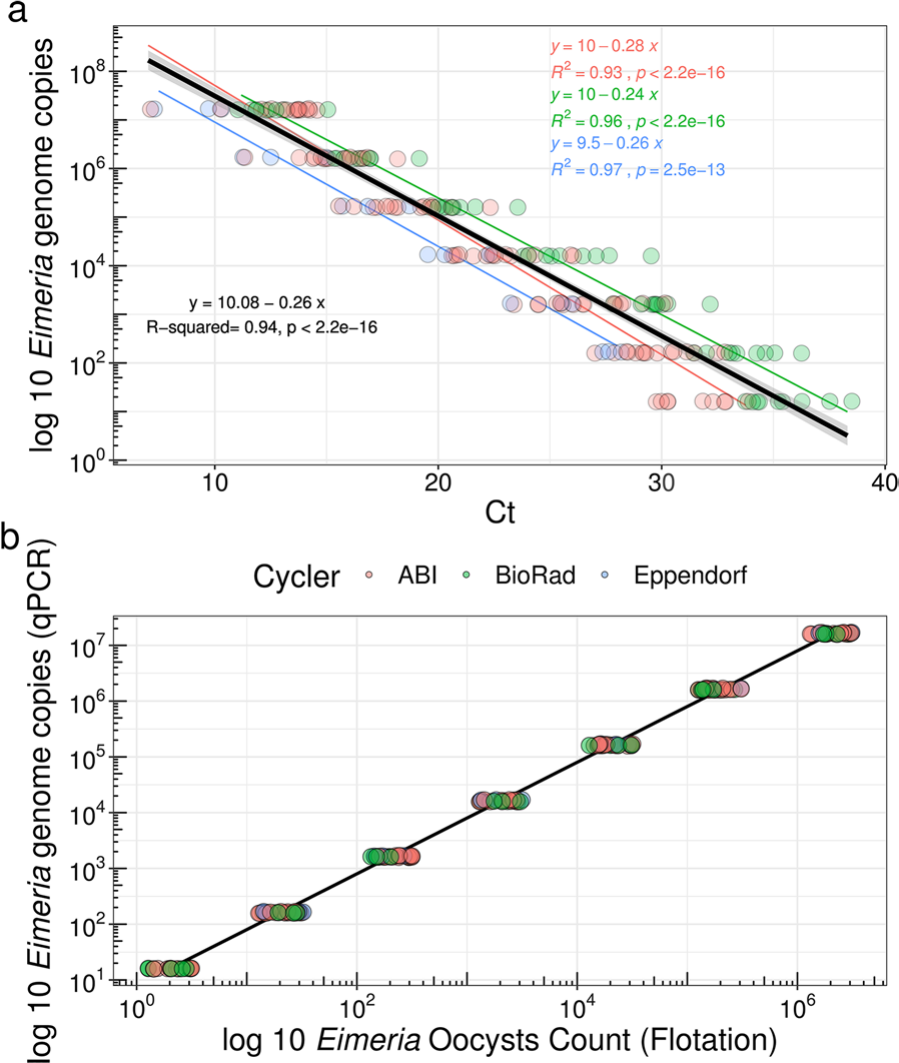
Development of an absolute qPCR quantification for *Eimeria* genome copy numbers. **a** Standard curves of *Eimeria* genomic DNA were generated from Ct values for *E. falciformis* and *E. ferrisi* DNA dilution series (*n* = 3) in the three cycling systems/instruments: ABI 7300 Real-Time PCR System (Applied Biosystems [ABI]; red circles), the MasterCycler® RealPlex2 cycler (Eppendorf; blue circles) and the CFX96™ Touch System (BioRad; green circles). Each point represents the linear decrease of the Ct values with increase of template along the curve. The linear equation, i.e. coefficient of determination (*R*^2^) is reported for each curve. **b** *Eimeria* genome copies per nanogram of gDNA are proportional to the counted oocysts with application of standard curve. Abbreviations: Ct, threshold cycle

We first performed a series of experiments to assure the accurate quantification of *Eimeria* DNA from faecal samples. We constructed standard curves from defined numbers of sporulated oocysts of *E. falciformis* and *E. ferrisi* using qPCR amplification of a fragment of the COI gene [16, 36]*.* Based on the known quantities of oocysts we estimated standard curves predicting the number of *Eimeria* genome equivalents from cycle thresholds. For the three qPCR cycling systems employed with the same DNA dilutions, we estimated technical variability in the present study to be 3–7% (*R*^2^ = 0.93 for the ABI7300 System, *R*^2^ = 0.96 for the CFX96™ cycler and *R*^2^ = 0.97 for the MasterCycler® RealPlex2 System; Fig. 1a). Significant differences among qPCR platforms were detected; therefore, a model with “qPCR platform” as an explanatory variable was used as a “standard curve” to predict *Eimeria* genome copies from Ct values (Fig. 1b).

To test the robustness of the approach, we additionally analysed (i) between-sample variation of different oocysts after flotation and sporulation and (ii) the impact of the “matrix” (faeces vs cleaned oocysts) used in the DNA extraction protocols on the efficiency of extraction and amplification. For this analysis we used DNA from previously floated oocysts, with different rates of sporulation and two different *Eimeria* species (*E. falciformis* in addition to *E. ferrisi*), to estimate the variation between different samples. Between-sample variation in *Eimeria* DNA content was 64.61%, but only 2.74% of this variation could be explained by differences in the proportion of sporulated versus unsporulated oocysts (Additional file 3: Fig. S2a; Additional file 4: Table S2). Amplification efficiency did not differ between *E. falciformis* and *E. ferrisi* preparations, meaning that our quantifications worked equally for both species (Additional file 4: Table S2).

To evaluate whether gDNA extraction from faeces impacts the quantification, we tested differences in the ratio of faecal material to oocysts. Extraction of humic substances carried over from the faecal DNA extraction could interfere with amplification. We confirmed that specific amounts of oocysts spiked into *Eimeria* DNA-free (uninfected) mouse faeces still produce a linear fit of standard curves comparable to that from oocyst flotations (Additional file 3: Fig. S2).

### *Eimeria* DNA can be detected earlier in the course of infection than oocysts

**Fig. 2:**
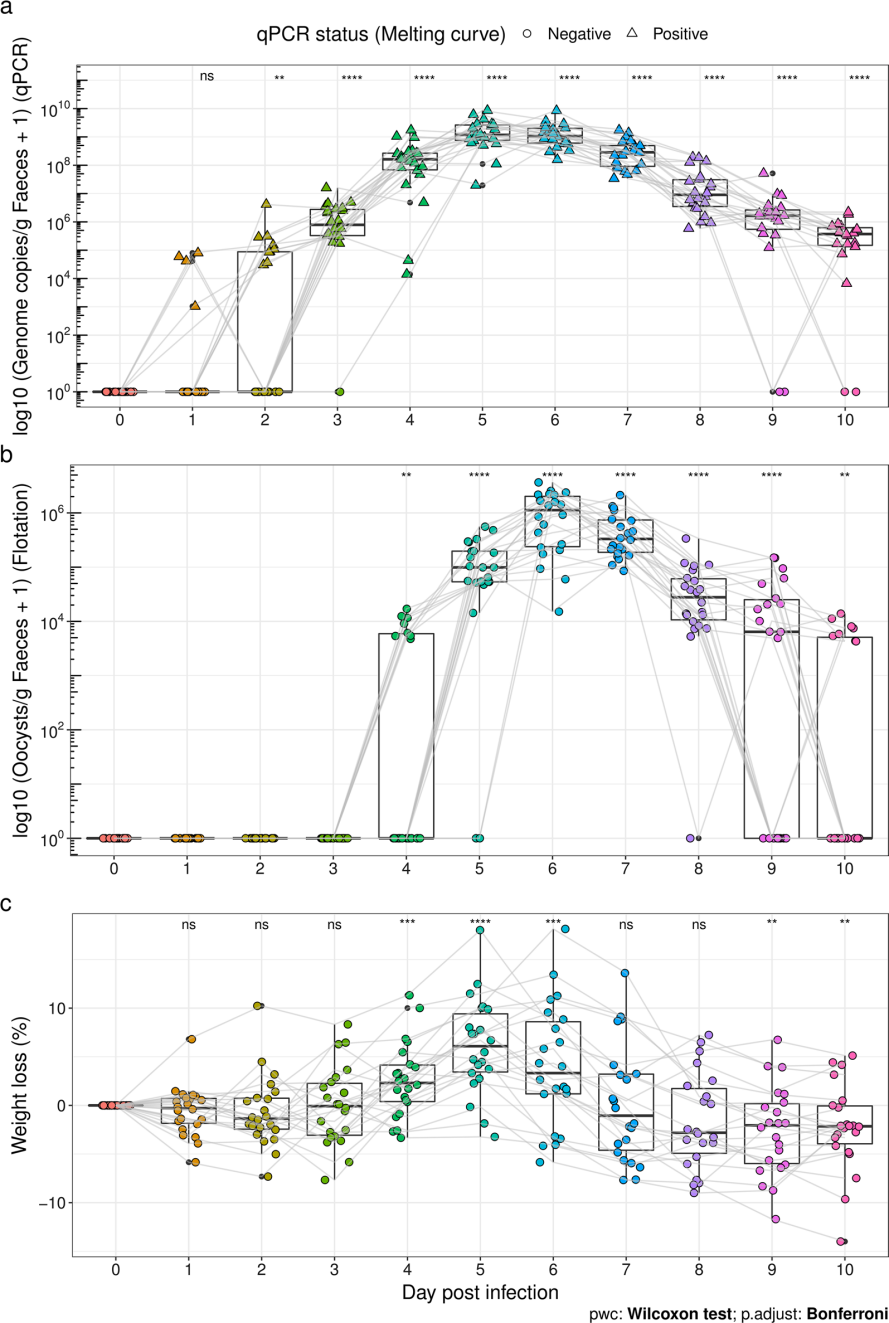
*Eimeria* quantification during the infection. Mice were infected with *E. ferrisi* (isolate Brandenburg64) and the infection course was tracked by: **a** *Eimeria* genome copies per gram of faeces estimated by qPCR, and** b** oocysts per gram of faeces estimated by flotation and counting. Note the different scales of the *y*-axes in these plots that indicate different orders of magnitude for both measurements, i.e. more genome copies than expected from the number of oocysts. In **a**, the shape represents the detection of *Eimeria* DNA in the faecal samples, as confirmed by melting curve analysis. **c** Weight loss during the infection relative to dpi 0. Boxplots represent the OPG or *Eimeria* genome copies per gram of faeces quantification for 22 mice during the infection. The centre line represents the median; box limits are the first and third quartiles; and the whiskers mark the interquartile range. Each filled circle represents an individual faecal sample (*n* = 242), colour-code by number of dpi. Grey lines link measurements for the same mouse individual. Statistical significance was evaluated against the same number of zero values with a Mann–Whitney U-test, and *P*-values were adjusted using Bonferroni correction. Asterisks indicate significance: **adjusted-*P* < 0.001, ***adjusted-*P* < 0.001. Abbreviations: ns, Not significant

Oocysts and *Eimeria* DNA in faeces were quantified during the first 10 dpi of house mice with *E. ferrisi*. Significant amounts of parasite DNA were detected as early as dpi 2 (two-sided Mann–Whitney U-test, *n* = 22,* U* = 30, *P* < 0.001, adjusted-*P* < 0.01) (Fig. 2a)—before the appearance of oocysts in faeces at dpi 4 (Fig. 2b). Based on OPG counts, we show the prepatent period to be 3 days and patency to last for 7 days, with a median maximum count at dpi 6 (Fig. 2b). We also tracked the weight loss during the experiment and observed a significant weight loss from dpi 4 to 6 compared to the beginning of infection (two-sided Mann–Whitney U-test, *n* = 22,* U* = 110, *P* < 0.001, adjusted-*P* < 0.001) (Fig. 2c).

### Oocyst counts and* Eimeria* DNA intensity correlate only weakly, especially early in infection when an overabundance of DNA is observed

**Fig. 3:**
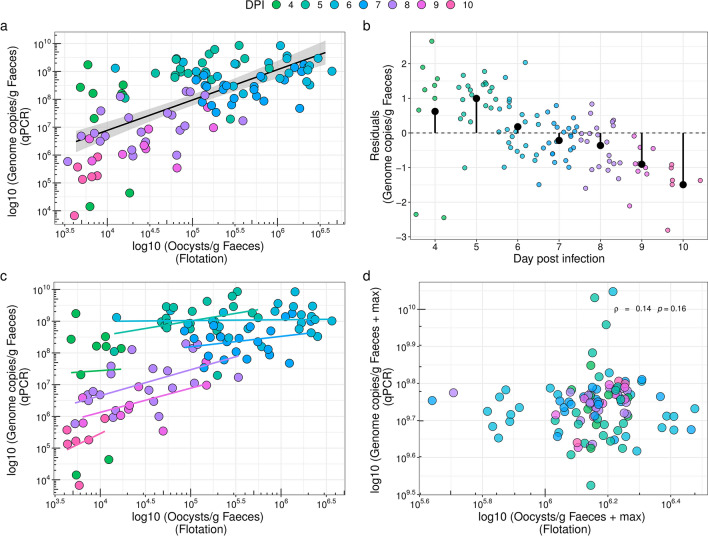
Quantitative relationship between genome copies per gram of faeces and oocysts per gram of faeces (OPG). **a** Relationship between genome copies per gram of faeces and OPG based on 109 non-zero observations from dpi 4 to 10 with *Eimeria* and the corresponding linear models. The expected number of genome copies for 1 oocyst fully sporulated should be around 8 (8 sporozoites per oocyst, should generate a difference of appox. 10^1^, at most). However, we consistently observed higher DNA intensities (of up to approx. 10^3^ more than OPG) for all but samples from dpi 8 and 9. **b** Residuals of the model in** a** ordered by dpi. Filled black circles indicate the mean per dpi. Early in infection, relatively more DNA is observed compared to oocysts, while later in infection it is relatively less. **c** Relationship between genome copies per gram of faeces and OPG with independent slope and intercept for each infection day (indicated by coloured lines). Positive slopes are only observed late in infection (on dpi 8 and 9) meaning that the number of oocysts partially explains the amount of DNA (only) at those time points. **d** The same relationship as shown in** c** using a time and individual demeaned dataset, in which genome copies and OPG were added to the maximum value for each mouse individual and time point before transformation with the common logarithm in order to remove within-individual and within-time-point correlations. As in **c** this demonstrates a very weak (non-significant) correlation between DNA and oocyst counts when time (and host individual) are considered

To determine whetheroocysts counted in faecal material are the sole source of *Eimeria* DNA, we modelled the first by the latter using a dataset containing non-zero observations for both measurements. We found that, when disregarding an effect of time (dpi), the number of OPG explains the number of *Eimeria* genome copies per gram of faeces (qPCR data) (*F*_(1, 107)_ = 79.36,* R*^2^_Adjusted_ = 0.4205, *β* = 1.0899, *P* < 0.001) (Fig. 3a).

We also observed that early infection (from dpi 4 to peak patency) was associated with positive residuals in this naïve model (Fig. 3b), while residuals after peak patency were negative or closer to zero. The result means that early in the infection, DNA was more abundant relative to oocysts, while this pattern reverted after the day of peak shedding.

The number of genome copies is established based on the known number of oocysts for a standard curve (Fig. 1), and an oocyst of *Eimeria* contains eight haploid sporozoites (fully sporulated). If oocysts were the predominant source of DNA, we would therefore expect eight genome copies per oocyst. Nevertheless, we observed early during the infection (before the peak of oocyst shedding) hundreds to thousands of times more DNA than explained by oocysts. Later during infection, this reduced to a 10- to 100-fold overabundance of DNA relative to oocyst counts. The overall correlation of DNA abundance and oocyst counts might be a trivial effect of both measures increasing over time. We thus tested whether the intensity of *Eimeria* DNA was explained by OPG within single time points during infection (dpi). This analysis resulted in non-significant slope terms for all dpi from dpi 4 to 10 after Bonferroni correction for multiple testing. For early dpi (4–7 dpi) the slope was virtually flat (*β*_dpi 4_ = 0.1959, *β*_dpi 5_ = 0.4764, *β*_dpi 6_ = 0.0248, *β*_dpi 7_ = 0.3292) (Fig. 3c).

To confirm this observation, we used a time and individual demeaned dataset. We observed no correlation between DNA and oocyst counts (Spearman’s ρ = 0.14, *n* = 109, *P* = 0.16). This analysis considered the time (and host individual) and corrects their statistical dependence by adding maximal values for time points and individuals. This procuder still results in a high power analysis, as replicates over time can be considered; nevertheless we detected no significant correlation between oocyst numbers and DNA intensity (Fig. 3d).

### *Eimeria* DNA in faeces is a better predictor of host health than *Eimeria oocyst* counts

**Fig. 4:**
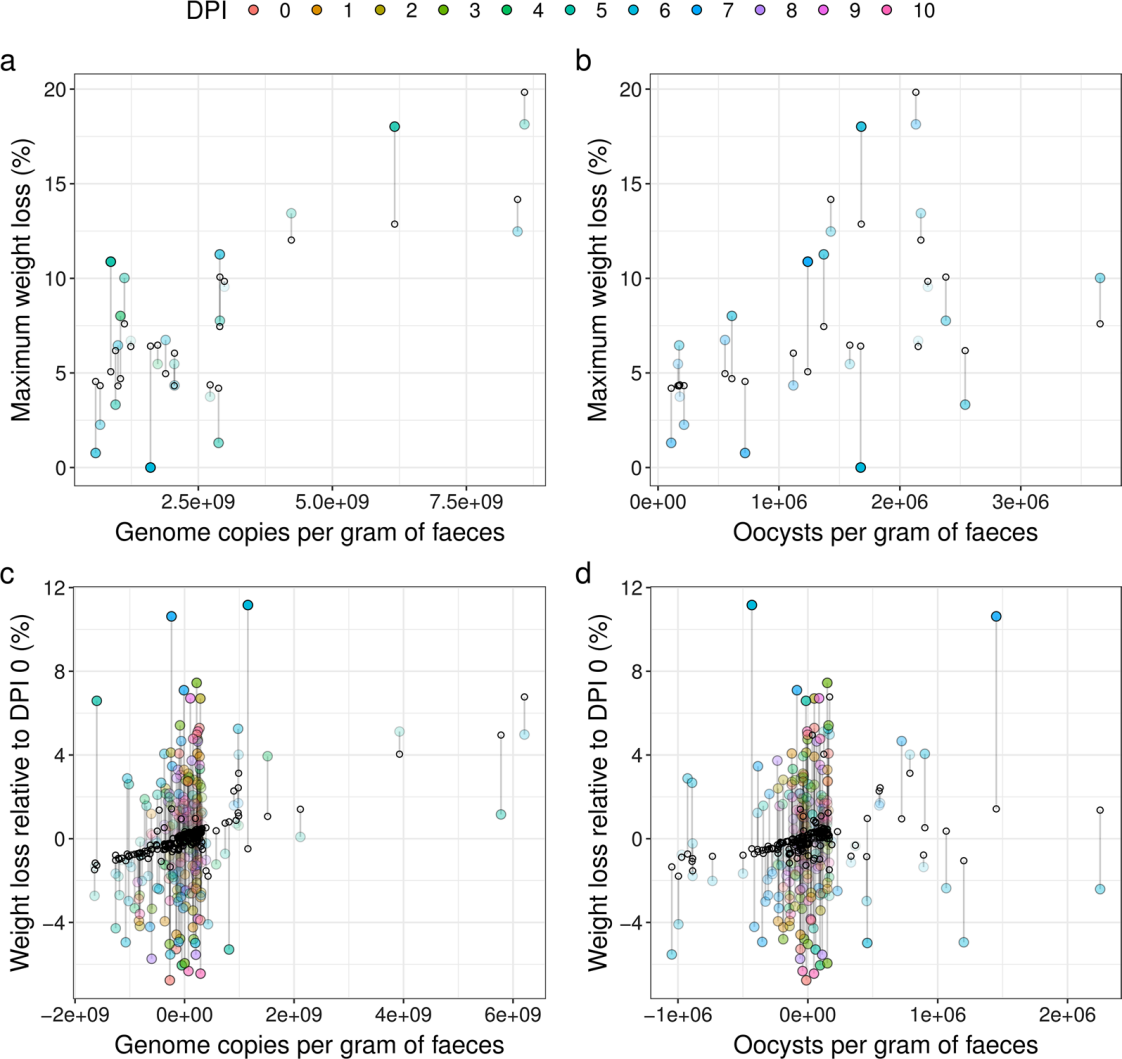
Host health is better explained by *Eimeria* DNA intensity than oocyst counts in faeces. Linear models for weight loss of 22 individuals over 10 dpi with *Eimeria.* Model A (*n* = 22): maximum weight loss predicted as interactions between genome copies per gram of faeces (**a**) and oocysts per gram of faeces (**b**), with each predictor plotted separately. Model B (*n* = 235): weight loss predicted as interactions between genome copies per gram of faeces (**c**) and oocysts per gram of faeces (**d**) using an individual and time (dpi) demeaned dataset, with each predictor plotted separately. Coloured circles represent the actual data, with the colours of the circles representing the day of maximum genome copies per gram of faeces (**a**), the day of maximum oocysts per gram of faeces (**b**) or the day of measurement (**c**,** d**). The white circles represent the predicted values. Transparency of the coloured points increases with proximity between measured and predicted points (smaller residuals). DNA intensity was found to be a stronger predictor of weight loss than OPG (Table 1), and the effect can be visualized as smaller residuals in **a** and **c** compared to **b** and **d**

To test whether the intensity of *Eimeria* DNA, oocyst counts or the combination of both are better predictors of host health, we used the maximally measured *Eimeria* DNA per gram of faeces and maximum OPG in a linear model trying to explain the weight maximally lost by an individual mouse (model A; LRT:* χ*^2^ = 22.44,* df* = 3, *P* < 0.001). As expected [7], we found that both OPG and faecal *Eimeria* DNA explained maximum weight loss during infection (LRT: OPG:* χ*^2^ = 6.66,* df* = 2, *P* = 0.036; genome copies per gram faeces:* χ*^2^ = 18.18, *df* = 2, *P* < 0.001). The interaction between the two terms was found to be not significant (LRT:* χ*^2^ = 2.54, *df* = 1, *P* = 0.11) (Table 1). The total proportion of variance explained by the model with the two predictors but without interaction was found to be 59.53%, with OPG contributing only 12.98% but faecal *Eimeria* DNA contributing 46.55%. After normalization, we found that the proportion of contribution of each predictor to the overall R^2^ is 78% for *Eimeria* genome copies per gram of faeces (Fig. 4a) and 22% for OPG (Fig. 4b). Additionally, we tested along the time-course of infection whether *Eimeria* DNA and oocyst counts predict the weight loss on particular dpi relative to dpi 0 using a time and individual demeaned dataset (model B; LRT:* χ*^2^ = 18.94, *df* = 3, *P* < 0.001). We found that DNA but neither OPG (genome copies per gram faeces:* χ*^2^ = 15.65, *df* = 2, *P* < 0.001; OPG:* χ*^2^ = 4.72, *df * = 2, *P* = 0.09) (Table 1), nor an interaction between the two explained weight loss. The total proportion of variance explained by the model including genome copies per gram of faeces (DNA) and OPG (without interaction) was 6.94, 5.71 and 1.22% of DNA and OPG contribution, respectively. After normalization, we found that the proportion of contribution of each predictor to the overall* R*^2^ is 82.30% for DNA (Fig. 4c) and 17.70% for OPG (Fig. 4d). Both models indicate that DNA intensity is a stronger predictor of weight loss than OPG. In addition, we used our DNA measurement to investigate differences in the correlation between parasite load and the impact on health by host genotype. We replicated the results of Balard et al. [7], showing that parasite load correlates positively with weight loss for *E. ferrisi* using our new DNA based measure. This means that resistance and tolerance are still uncoupled for this parasite when DNA intensity is used to assess resistance (Additional file 5: Fig. S3).

**Table 1:** Predicted models of host health by *Eimeria* DNA and oocysts in faeces

Models	Estimate	Standard error	*t*-value	*P*-value	* χ*^2^	*df *	*P*-value
Model A: Maximum weight loss							
Intercept	4.30	2.32	1.85	0.08			
Genome copies per gram of faeces	− 1.37 × 10^–10^	1.12 × 10^–9^	− 0.12	0.90	18.18	2	< 0.001***
OPG	− 9.44 × 10^–8^	1.34 × 10^–6^	− 0.07	0.94	6.66	2	0.036*
Genome copies × OPG	9.24 × 10^–16^	6.22 × 10^–16^	1.48	0.15	2.54	1	0.11
Model B: Weight loss with time and individual demeaned dataset							
Intercept	− 5.27 × 10^–3^	0.19	− 0.03	0.98			
Genome copies per gram of faeces	7.97 × 10^–10^	2.57 ×^−10^	3.01	< 0.001	15.65	2	< 0.001***
OPG	1.48 × 10^–6^	6.93 × 10^–7^	2.14	0.03	4.72	2	0.09
Genome copies × OPG	1.52 × 10^–15^	1.08 × 10^–15^	1.42	0.16	2.03	1	0.15

The parameter estimates are reported with the corresponding standard error,* t*-statistics and the corresponding *P*-value, and the * χ*^2^ test for each explanatory variable with the corresponding *P*-value

Significant difference at *P < 0.05; **P < 0.01; ***P < 0.001

## Discussion

Parasite load can be defined and measured differently, and those measurements might have distinct meaning for the biology of the host and the parasite. In this study, we compared the quantification of parasite load using qPCR and coprological counting of transmission stages of the parasite (oocysts). We found that the number of oocysts inadequately explained the amount of parasite DNA in host faeces in the same sample. We show in the *E. ferrisi* – *M. musculus* system that faecal DNA content is a better predictor of a health effect of the parasite infection on the host than oocyst counts. In addition, we argue that DNA-based measurements of intensity are biologically different and complementary indicators of parasite load in this system, and support their independent assessment in general.

We show an increased sensitivity of the qPCR approach with a mitochondrial multi-copy gene to detect *Eimeria* in our system. We quantified more parasite DNA compared to oocysts and recovered more positive samples during infection. The latter is not surprising, as previous studies of both helminths and protozoans showed that parasite detection is more sensitive by qPCR than by coprological counting methods [37, 38]. Studies in bacteria (*Mycobacterium tuberculosis* and *Escherichia coli*) and parasites (*Plasmodium falciparum* and *Toxoplasma gondii*) support the use of multi-copy genes to increase the sensitivity of detection and quantification by qPCR compared to the use of a single-copy gene or light microscopy, even when the specificity might be slightly reduced [39–41].

Using an SYBR green-based protocol could lead to the detection of unspecific signals at late cycles. Here, we were able to identify all samples with amplification noise at dpi 0 as “melting curve negative” by performing melting curve analysis on the samples blinded and randomized. Likewise, no-template controls in the standard curve analysis could all be identified in a blinded assessment (Additional file 2: Fig. S1b). This demonstrates high specificity. In four samples we had “melting curve positive” detecting *Eimeria* DNA at dpi 1 (Fig. 2a), meaning that we might detect oocysts from the infection inoculum passing through the mouse on this day. We believe that our melting curve analysis approach can reach the sensitivity and specificity of TaqMan assays to quantify parasites, as demonstrated earlier for *Leishmania* in humans [42]. In contrast, a relatively high detection limit is usual when counting oocysts from a subset of the flotation of faecal material.

The intensities of *E. ferrisi* quantified using faecal DNA reflect a similar chronology to oocyst counts for this parasite in our study, as has been previously described [15, 18, 22]. Nevertheless, we detected parasite DNA earlier than oocysts in the course of an experimental infection. Noting this shifted chronological pattern of intensities inferred from oocysts and DNA against each other, we further asked whether an increased sensitivity is the only reason for disagreement between oocyst and DNA-based assessments.

Parasite DNA in host faeces derived from stages other than oocysts might result in disagreement between the two measures. While we observed relatively high amounts of DNA compared to oocysts at 4, 5 and 6 dpi, this pattern changed at later dpi (8, 9 and 10 dpi) when we quantified more oocysts than DNA. Additionally, qPCR detected 10- to 1000-fold more DNA than expected from the oocysts counted in the sample alone, meaning that sources other than oocysts contribute *Eimeria* DNA, including sporozoites, merozoites and gametocytes failing to invade cells or schizonts from apoptotic cells expelled with the faeces. In poultry *Eimeria*, the induction of NF-k*β* inhibits host cell apoptosis early in infection, while in later stages, apoptosis is rather promoted to facilitate the release of the parasite from cells [43–46]. DNA from non-viable parasites in the faeces could thus be a combined result of both parasite replication and (for individual hosts more or less successful) host response against the infection [47, 48]. The oocyst output of *Eimeria* decreases output when the inoculation dose in an immunologically naive host is increased over a certain threshold [49]. It could be that this so-called crowding effect (and reaching of the respective crowding threshold) leads to expulsion of immature stages for which we measure only DNA but do not count oocysts. We conclude that, especially at early dpi, DNA in faeces derives from *Eimeria* stages other than oocysts.

Based on these differences in DNA/oocyst ratios along the temporal progression of the infection, we propose that DNA in faeces could be employed to determine the chronological state of natural infections when both measurements are available, with a high amount of DNA relative to oocysts indicating an early infection. Nevertheless, we note that for this purpose the DNA/oocyst ratio during infection should be determined for each species in question. It might then be possible to determine the timing of infection in naturally infected wildlife or livestock using the DNA/oocyst ratio for other *Eimeria* species.

The number of oocysts in a sample at a particular dpi is a poor indicator of the amount of DNA recovered, and vice versa. The discrepancy between the two measurements leads to the question whether they both reflect different but meaningful characteristics of the host–parasite system. Without question, oocyst counts are the more meaningful measure of parasite load when reproductive and overall fitness of the parasite should be studied [50, 51]. Counts of transmissive stages are certainly a more relevant component of parasite fitness than DNA, deriving from potentially expelled non-transmissive stages, which might even indicate the inefficient performance of the parasite. For unsporulated oocysts, either different DNA extraction efficiency [52] or lower genome copy number (2 genome copies in unsporulated vs 8 genome copies in sporulated oocysts) or the combination of both could explain deviations from genome copy numbers predicted by oocyst counts. However, we dismissed this with our analysis.

It is less clear how other conclusions on the host–parasite system’s biology can be drawn from the two measurements. One such characteristic, resistance, i.e. the capacity of the host "to limit parasite burden” [53], is best studied in this context. Resistance of hosts is usually quantified as the inverse of parasite load [54]. Which measure best captures resistance (or—in other words—which of these subtly distinct resistance phenotypes is more relevant): DNA intensity reflecting asexual expansion or oocysts reflecting reproduction?

When mitigation of the health effect of an infection and resistance are positively correlated, the strength of this correlation should allow a decision for the better measurement of resistance [55, 56]. In *E. ferrisi* infection of different house mice genotypes, this is the case, as higher resistance (lower oocyst count) means better health of the host, as previously described by Balard et al. ([7]; Additional file 5: Fig. S3). We used two different approaches to analyse this: first, we tested whether the maximum potential resistance measure (DNA intensity or oocyst counts) could predict maximal weight loss; second, we evaluated which is a better predictor for health (weight loss) on individual days. In both cases, DNA intensity was a better predictor of host health, as higher faecal DNA intensities were associated with higher weight loss.

We alternatively had hypothesized that shedding additional DNA with earlier parasite stages could be a result of parasite killing and contribute positively to a health effect. On the contrary, however, the higher amounts of DNA early in infection did not positively impact the host’s health, rather there was a negative impact. We conclude that in the *E. ferrisi*–*M. musculus* system, asexual expansion of the parasite can be assessed directly as tissue DNA intensity ([15]; a measurement for which the hosts need to be sacrificed) or via faecal DNA intensity. In both cases, DNA is likely to be the more relevant measure of host resistance (or the lack thereof, in the case of high intensities) than oocyst counts, as an early expansion of *Eimeria* needs to be resisted by the host to achieve positive health outcomes. In a previous work, we observed that *E. ferrisi* induced harm earlier also relative to the patent period and oocyst shedding than other species (*E. falciformis*) [7, 22], possibly indicating that *E. ferrisi* causes harm by its asexual expansion rather than by its oocyst production. It could indeed be that other species (e.g. *E. falciformis* in our mouse host) would cause harm later, and for these species the relationship of DNA to oocyst output might differ. Further studies are needed to assess faecal DNA/oocyst ratios in other *Eimeria* species to address the differences between parasites with different virulence. This will allow us to to determine for other species whether faecal DNA or oocysts are more closely correlated to health effects of the infection.

## Conclusions

We here show that the amount of coccidian parasite DNA in host faeces provides information different and complementary to counts of transmissive oocyst stages. In our example, DNA-based quantification of parasite loads can predict the parasite’s impact on host health better than the classical oocyst counts. In general, DNA-based “molecular quantification” of parasite load should not necessarily be validated against classical coprological approaches. Instead, information from both molecular and classical coprological techniques should be assessed for the complementarity of information they can provide.

## Supplementary Information

**Additional file 1: Table S1.** General characteristics of the cohort of mice employed during the infection experiment.

**Additional file 2: Figure S1. a** We observed that some NTC presented an unspecific signal leading to high Ct values that might get confused with samples containing low quantities of *Eimeria* DNA. **b** In addition to the Ct estimation, melting curve analysis allowed us to distinguish true *Eimeria* amplifications by establishing a threshold of Tm based on the standards and positive controls.

**Additional file 3: Figure S2. a** The intersample variation between oocyst count and *Eimeria* genome copies estimation by qPCR (*n* = 19). **b** Comparison of oocysts and genome copies in DNA from mock samples (*n* = 8) (blue) and standard curve (red). Genome copies estimations using gDNA from faeces spiked with sporulated oocysts correspond to 1.8-fold reduction compared to qPCR estimations using gDNA from sporulated oocysts.

**Additional file 4: Table S2.** Linear model to assess the intersample variation.

**Additional file 5: Figure S3.** Non-significant positive correlation between mean maximum parasite load (left: oocysts per gram of faeces/right: *Eimeria* genome copies per gram of faeces) and mean relative weight loss. Absence of correlation between maximum oocysts per gram of faeces or genome copies per gram of faeces used as a proxy for (inverse of) resistance and tolerance; grey error bars represent 95% confidence intervals. Our results do not support coupling between resistance and tolerance for *Eimeria ferrisi* independently of the parasite load measured that is employed.

## Data Availability

All data generated and code used for the analysis during this study are publicly available in https://github.com/derele/Eimeria_Quant and its additional files.

## Abbreviations

*df*: Degrees of freedom
dpi: Day(s) post-infection
HMHZ: House mouse hybrid zone
LRT: Likelihood ratio test
OPG: Oocyst per gram
qPCR: Quantitative PCR
