# Supplementary material for: DNA-based quantification and counting of transmission stages provides different but complementary parasite load estimates: an example from rodent coccidia (Eimeria)
Source: Parasit Vectors. 2022 Feb 4;15:45. doi: 10.1186/s13071-021-05119-0 (PMC8815199; doi:10.1186/s13071-021-05119-0)
Supplement: Supplementary file 1 — Additional file 1: Table S1. General characteristics of the cohort of mice employed during the infection experiment. [file 13071_2021_5119_MOESM1_ESM.pdf]

Table S1. General characteristics of the cohort of mice employed during the infection experiment

Identification code	Weight at DPI 0 (g)	Age at DPI 0 (weeks old)	Sex	Genome	Strain
LM0204	19.26	15.43	M	mus x mus	PWD_BUSNA
LM0206	16.97	11.43	F	mus	BUSNA_BUSNA
LM0207	17.27	17.71	M	mus	PWD_PWD
LM0208	22.82	15.71	M	dom	STRA_SCHUNT
LM0209	22.09	13.86	F	dom	STRA_STRA
LM0210	15.65	9.00	M	mus x dom	PWD_SCHUNT
LM0211	17.42	14.14	M	mus	PWD_PWD
LM0212	21.58	12.86	M	dom	SCHUNT_STRA
LM0213	15.39	17.71	F	mus	PWD_PWD
LM0214	27.43	13.86	M	dom	STRA_STRA
LM0215	23.9	19.43	F	mus x dom	BUSNA_STRA
LM0216	18.37	10.57	F	mus	BUSNA_BUSNA
LM0217	17.74	10.57	M	mus	BUSNA_BUSNA
LM0218	20.36	9.00	M	mus x dom	PWD_SCHUNT
LM0219	19	15.43	M	dom x mus	SCHUNT_PWD
LM0220	19.72	14.14	F	mus x mus	BUSNA_PWD
LM0221	19.73	9.86	M	dom	SCHUNT_SCHUNT
LM0222	23.99	17.71	M	dom x mus	STRA_BUSNA
LM0223	20.73	12.43	M	dom	SCHUNT_SCHUNT
LM0224	19.21	14.14	M	mus x mus	BUSNA_PWD
LM0225	28.05	13.86	M	dom	STRA_STRA
LM0226	15.16	12.43	F	dom	SCHUNT_SCHUNT
