# Supplementary figures and images for: DNA-based quantification and counting of transmission stages provides different but complementary parasite load estimates: an example from rodent coccidia (Eimeria)
Source: Parasit Vectors. 2022 Feb 4;15:45. doi: 10.1186/s13071-021-05119-0 (PMC8815199; doi:10.1186/s13071-021-05119-0)

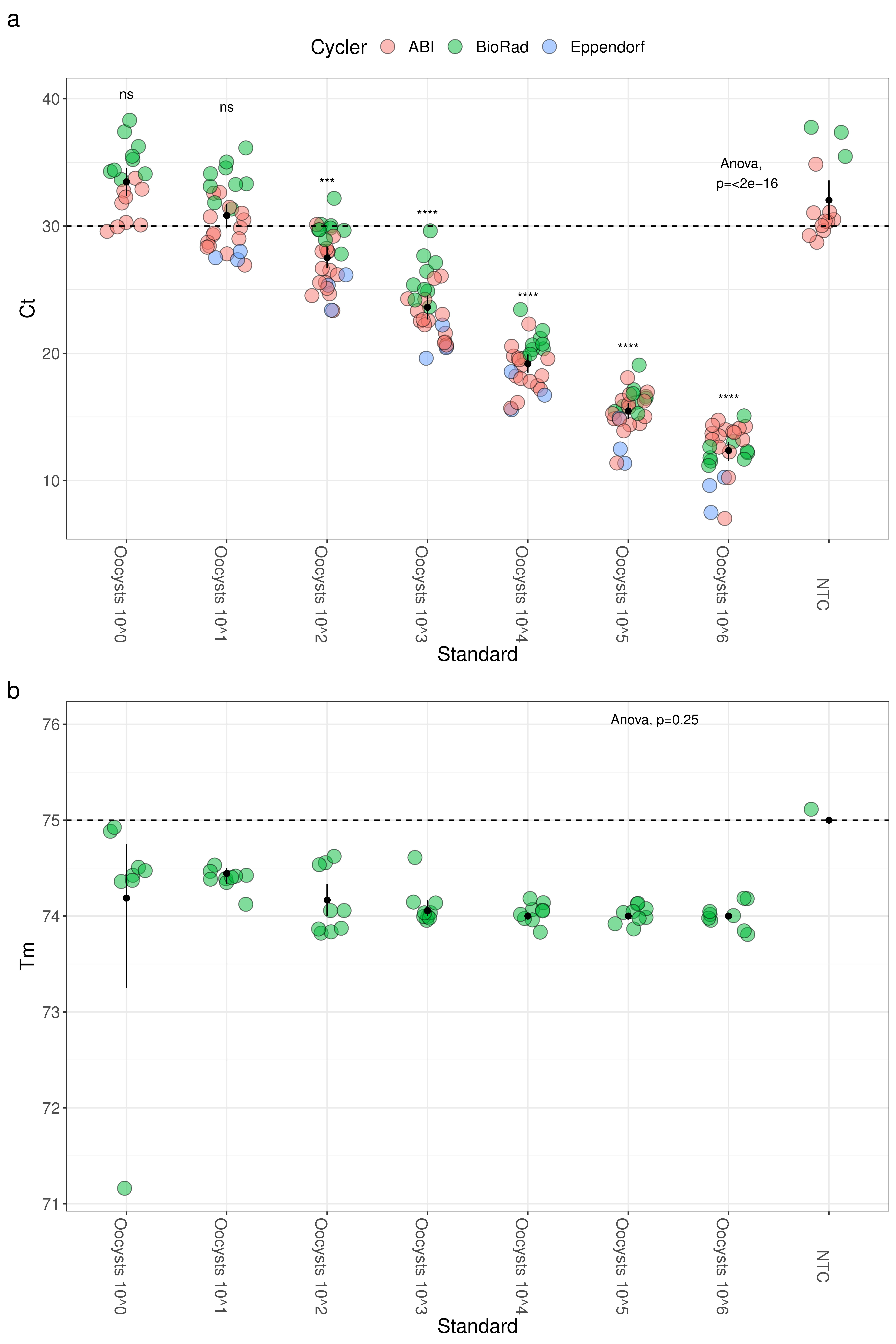

Supplement: Supplementary file 2 — Additional file 2: Figure S1. a We observed that some NTC presented an unspecific signal leading to high Ct values that might get confused with samples containing low quantities of Eimeria DNA. b In addition to the Ct estimation, melting curve analysis allowed us to distinguish true Eimeria amplifications by establishing a threshold of Tm based on the standards and positive controls. [file 13071_2021_5119_MOESM2_ESM.tiff]

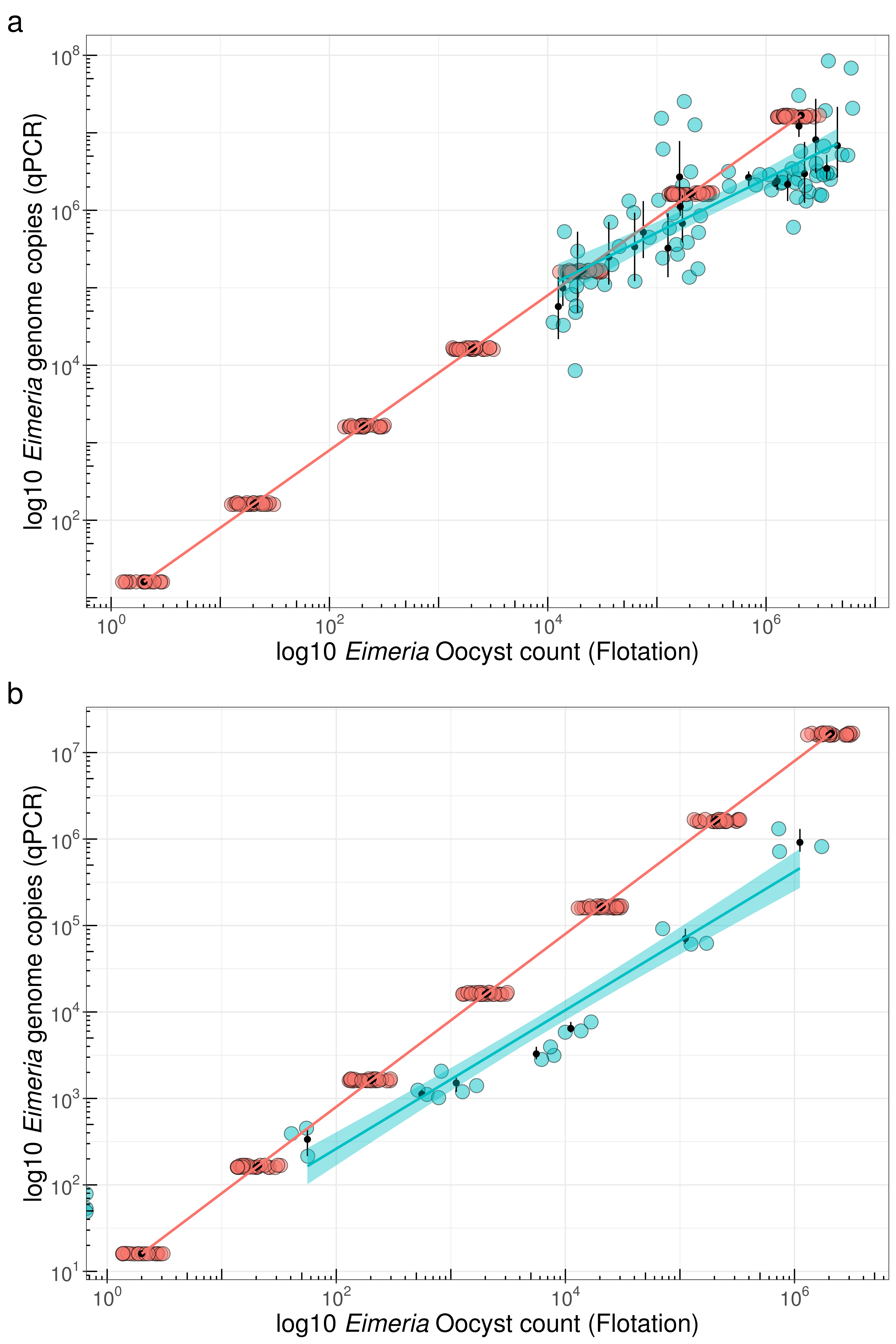

Supplement: Supplementary file 3 — Additional file 3: Figure S2. a The intersample variation between oocyst count and Eimeria genome copies estimation by qPCR (n = 19). b Comparison of oocysts and genome copies in DNA from mock samples (n = 8) (blue) and standard curve (red). Genome copies estimations using gDNA from faeces spiked with sporulated oocysts correspond to 1.8-fold reduction compared to qPCR estimations using gDNA from sporulated oocysts. [file 13071_2021_5119_MOESM3_ESM.tiff]

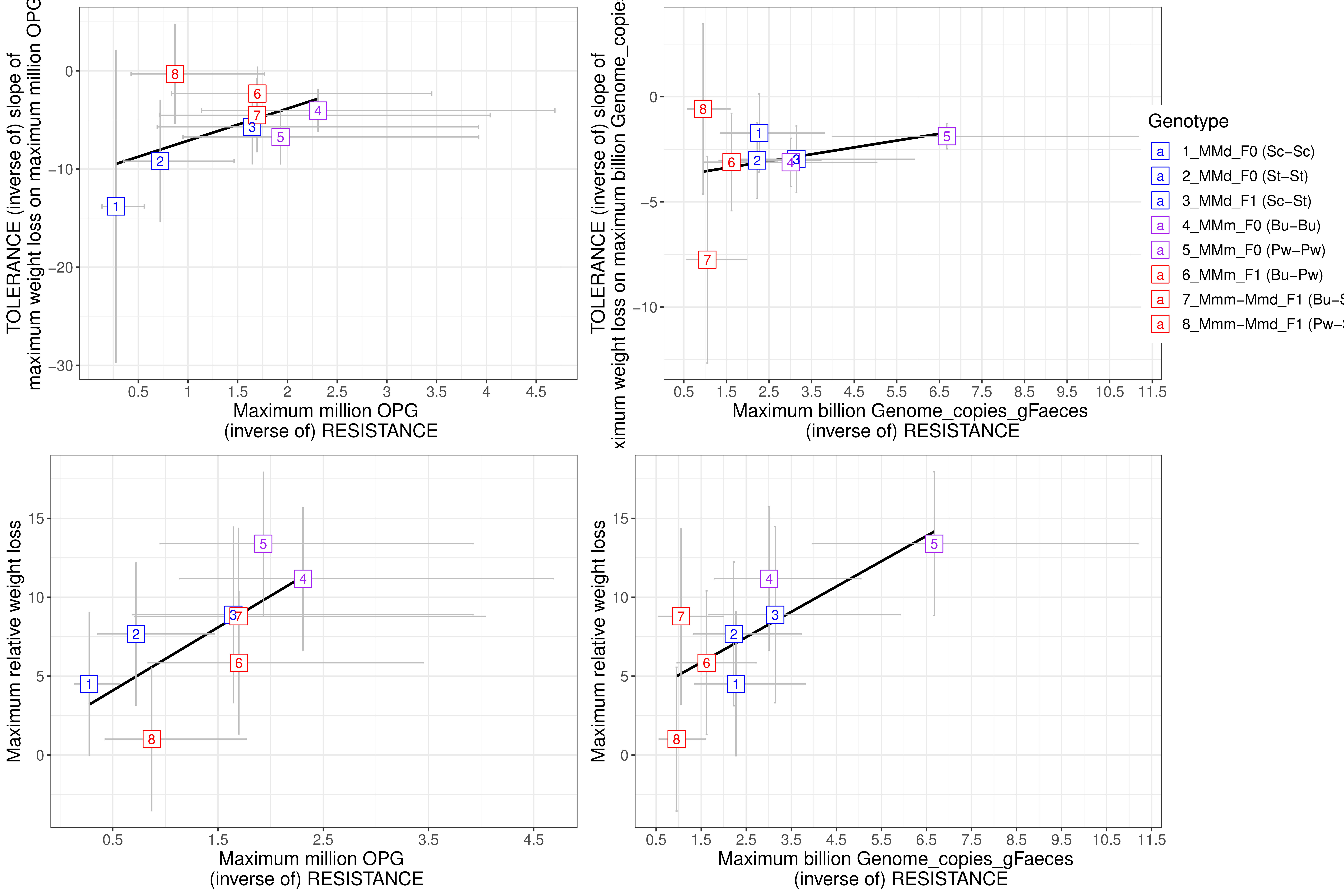

Supplement: Supplementary file 5 — Additional file 5: Figure S3. Non-significant positive correlation between mean maximum parasite load (left: oocysts per gram of faeces/right: Eimeria genome copies per gram of faeces) and mean relative weight loss. Absence of correlation between maximum oocysts per gram of faeces or genome copies per gram of faeces used as a proxy for (inverse of) resistance and tolerance; grey error bars represent 95% confidence intervals. Our results do not support coupling between resistance and tolerance for Eimeria ferrisi independently of the parasite load measured that is employed. [file 13071_2021_5119_MOESM5_ESM.tiff]
