# Supplementary material for: DNA-based quantification and counting of transmission stages provides different but complementary parasite load estimates: an example from rodent coccidia (Eimeria)
Source: Parasit Vectors. 2022 Feb 4;15:45. doi: 10.1186/s13071-021-05119-0 (PMC8815199; doi:10.1186/s13071-021-05119-0)
Supplement: Supplementary file 4 — Additional file 4: Table S2. Linear model to assess the intersample variation. [file 13071_2021_5119_MOESM4_ESM.pdf]

Table S2. Linear model to assess the intersample variation

Model<br>log10(Genome copies) ~ log10(Oocyst count) + Parasite + Cycler + Sporulation rate					
	**Estimate**	**Std. Error**	**T value**	**Pr(>|t|)**	**Explained variation**
Intercept	2.586812	0.431889	5.990	8.18E-08***	
**Log10 (Oocyst counts)**	0.599419	0.080232	7.471	**1.72E-10*** **	**58.41%**
Parasite	-0.397490	0.219253	-1.813	0.0741	0.58%
Cycler	0.452336	0.304046	1.488	0.1413	2.87%
**Sporulation rate**	0.003753	0.001611	2.330	**0.0227***	**2.74%**
